# Abcès hypophysaire chez un hémodialysé chronique traité médicalement: à propos d'une observation

**DOI:** 10.11604/pamj.2015.20.107.5838

**Published:** 2015-02-05

**Authors:** Yassir Zajjari, Faycal El Guendouz, Ali Akhaddar, Mohamed Benyahia

**Affiliations:** 1 Service de Néphrologie et d'Hémodialyse, Hôpital Militaire d'Instruction Mohammed V, Rabat, Maroc; 2 Service d'Endocrinologie Hôpital Militaire d'Instruction Mohammed V, Rabat, Maroc; 3 Service de Neurochirurgie Hôpital Militaire d'Instruction Mohammed V, Rabat, Maroc

**Keywords:** Abcès hypophysaire, hémodialyse, antibiotiques, Pituitary abscess, hemodialysis, antibiotics

## Abstract

L'abcès hypophysaire est une pathologie rare et fatale en absence de traitement adéquat. Il est à évoquer devant un tableau clinique d'hypertension intracrânienne, une dysrégulation hormonale hypophysaire et un contexte infectieux. Une conduite thérapeutique basée sur une antibiothérapie pourrait être tenté si diagnostic précoce. Nous rapportons un cas d'abcès hypophysaire survenant chez un hémodialysé chronique pris en charge par une antibiothérapie seule dont l’évolution était favorable avec un recul de 2 ans.

## Introduction

L'abcès hypophysaire est une pathologie rare et fatale en absence de traitement adéquat [1]. Nous rapportons un cas d'abcès hypophysaire survenant chez un hémodialysé chronique pris en charge par une antibiothérapie seule.

## Patient et observation

**Figure 1:**
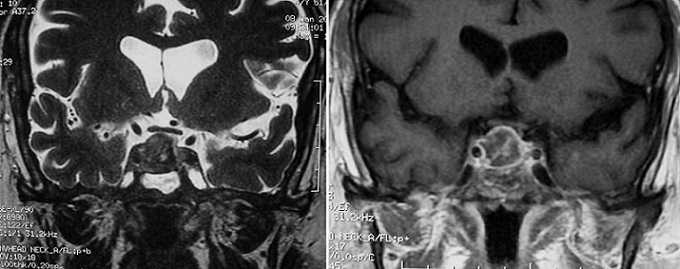
IRM cérébrale d'une coupe coronale en T2 montrant un processus hypophysaire avec prise de contraste périphérique en faveur d'un abcès

Un homme âgé de 49ans, hémodialysé chronique depuis 2005, suite à une maladie de Berger, qui a consulté pour des céphalées fébriles, associés à une diplopie horizontale droite, avec à l'examen une paralysie du III droit extrinsèque et sans syndrome méningé. Le bilan biologique a objectivé un syndrome inflammatoire, la ponction lombaire réalisée après une TDM cérébrale sans anomalies était normale. L'IRM cérébrale a montré un processus intrasellaire de 21 x 20mm, associé à une sinusite sphénoïdale (Figure 1). L'hypophysiogramme réalisé par la suite était en faveur d'un panhypopituitarisme. Le traitement institué en urgence comportait une antibiothérapie parentérale probabiliste à large spectre à base de ceftriaxone et de ciprofloxacine poursuivie pendant 12 semaines, associée à une opothérapie substitutive. L’évolution clinique et biologique était favorable avec un recul de 2 ans.

## Discussion

La première description d‘abcès hypophysaire remonte à 1914, avec depuis une centaine de cas répertoriés dans la littérature [1]. Classiquement il se manifeste par un syndrome tumoral, un syndrome infectieux et un tableau d'insuffisance antéhypophysaire [2]. Dans 10% des cas il survient sur un terrain particulier, l'infection se fait le plus souvent par contiguïté à partir d'une sinusite ou par voie hématogène [3]. Dans notre cas l'abcès hypophysaire semble être favorisé par le terrain d'immunodépression (hémodialyse chronique), et l'infection est secondaire à une sinusite sphénoïdale. La sémiologie IRM est non spécifique (en T1 l'abcès est hypo ou isointense, en T2 hyperintense et présente une prise de contraste annulaire), mais elle permet de le suspecter et d'indiquer une chirurgie en urgence [4]. Habituellement le traitement associe une antibiothérapie à la chirurgie. Cependant dans la plus grande série de la littérature rapportant 33 cas d'abcès hypophysaire, seulement trois patients ont été traités par une antibiothérapie seule qui était suffisante vu la précocité du diagnostic [5]. Des rares auteurs ont rapporté également des succès thérapeutiques avec une antibiothérapie seule [6]. Dans notre cas l’évolution clinique et biologique de l'abcès hypophysaire était favorable sous antibiothérapie et hormonothérapie substitutive épargnant au malade les risques d'une chirurgie laborieuse vu son terrain.

## Conclusion

Malgré sa rareté, l'abcès hypophysaire doit être évoqué devant un tableau clinique d'hypertension intracrânienne, une dysrégulation hormonale hypophysaire et un contexte infectieux. Une conduite thérapeutique basée sur une antibiothérapie pourrait être tenté si diagnostic précoce. Dans notre cas l'abcès hypophysaire semble être favorisé par le terrain d'immunodépression (hémodialyse chronique) et l’évolution était favorable sous antibiothérapie et hormonothérapie substitutive avec un recul de 2 ans.
